# The use of dried blood spot cards to assess serologic responses of individuals vaccinated against measles, hepatitis A, tetanus, influenza and varicella zoster

**DOI:** 10.1371/journal.pone.0265813

**Published:** 2022-03-24

**Authors:** Brian Reinhardt, Robert Taylor, Colin Dawkins, Taylor Banks, Nora Watson, Appavu Sundaram, Daniel Ewing, Janine Ruth Danko

**Affiliations:** 1 Department of Research Programs, Walter Reed National Military Medical Center, Bethesda, Maryland, United States of America; 2 Henry Jackson Foundation, Bethesda, Maryland, United States of America; 3 Department of Allergy/Immunology, Naval Medical Center Portsmouth, Portsmouth, Virginia, United States of America; 4 Department of Viral and Rickettsial Diseases, Naval Medical Research Center, Silver Spring, Maryland, United States of America; 5 Infectious Disease Service, Walter Reed National Military Medical Center, Bethesda, Maryland, United States of America; Stanford University School of Medicine, UNITED STATES

## Abstract

Traditional blood sampling by venipuncture is cumbersome and relatively expensive. Dried blood spot (DBS) sampling is desirable because of its ease of sample collection, transportation and storage. It has been used in clinical diagnosis but not been thoroughly studied for the potential use to assess the immune status of individuals following natural infection or preventive vaccination. The goal of this study was to compare DBS to traditional blood samplings in detection of antibodies in individuals vaccinated against measles, hepatitis A, tetanus, influenza and varicella zoster. Enzyme linked immunosorbent assay (ELISA) was used to test DBS eluates and serum samples for antibodies against measles, varicella, tetanus and hepatitis A. Sensitivities, specificities, and correlation coefficients were evaluated to compare optical density (OD) values of paired serum and DBS samples. The long-term stability of DBS samples at different temperatures was assessed using simulated immune measles blood. DBS OD was highly correlated with serum OD for antibodies to measles (r = 0.93), varicella (r = 0.82), and tetanus (r = 0.91). Sensitivities of DBS OD ranged from 86–99% and specificities ranged from 96–100% using cut-offs established by each assay. By contrast, the hepatitis A data showed a low sensitivity (31%) and weak correlation (r = 0.14) between DBS and serum samples. Antibody titers in serum samples for anti-influenza A (H1N1 and H3N1) failed to correlate in DBS eluates in HAI and MN assays. DBS samples were stable for 4 weeks when stored at room temperature and for 6 months at 4°C. DBS sampling was sensitive, specific, and highly correlated with traditional venipuncture sampling in detection of antibodies against measles, tetanus and varicella zoster, but not hepatitis A and influenza. Thus, the success of using DBS sampling to assess the antibody levels in immunized individuals may be dependent on the pathogens and the development of the assay used.

## Introduction

Understanding a particular infectious disease burden or vaccine coverage *in situ* in a particular region is a critical part of eliminating the disease, decreasing the disease burden and to determine the need for mass vaccinations during an outbreak. Screening and surveillance activities make up the core principles of these investigations. Sampling individual serologies by venipuncture represents the gold-standard in most health systems, hospitals and treatment facilities. These methods require significant numbers of experienced human resources, equipment, cold-chain storage and infrastructure [1, 2]. Additionally, traditional serum sampling is expensive, invasive and not amenable to large scale assessments or screenings. The collection, processing and transportation to a testing laboratory of a large numbers of clinical samples present significant cost and logistic issues.

An alternative to blood draws for serological testing is dried blood spots (DBS), in which blood from a finger prick is dripped onto marked circles on a filter paper card. Application of DBS dates back to the early 1960s when Dr. Robert Guthrie first began collecting heel-prick, blood spot samples from newborns to detect phenylketonuria [3]. Dried blood spots are widely applied in numerous bioanalytical assays and have gained a significant role in the screening of inherited metabolic diseases, in pharmacokinetic and pharmacodynamic modeling, and at each step of the therapeutic cascade of HIV, hepatitis B and hepatitis C: screening, confirmation, measurement of the replication, and in the analysis of individual therapeutic failures [4, 5]. DBS have also been successfully used in diagnostic testing for measles, hepatitis E, Ebola and polio [6–12]. The use of DBS in HIV viral load monitoring has also been reported in the last decade [13, 14].

The procedure for DBS collection has been previously detailed [15]. The DBS card is labeled at the time of the collection with patient and study-identifying information, and this information remains with the specimen until testing is initiated, decreasing the opportunity for mislabeling. There is no need for experienced technicians; the blood is easily collected by pricking a finger or heel (convenient for collecting blood from babies and small children). In addition, samples are dried and stored at room temperature, occupy little space, are easy to store or mail, cannot be broken or spilled and can be readily transported [16]. Importantly, many analytes, including antibodies, are stabilized once dried on filter paper, despite given fluctuations in environmental conditions, such as shipping temperatures. DBS-based tests offer several advantages over serum testing when resource and environmental conditions are challenging. DBS collection is less expensive, relies on more portable equipment, and can be done effectively by a minimally trained individual. DBS specimens collected outside of healthcare facilities can also be considered as an alternative to rapid diagnostic tests (RDTs).

There are limited studies using this method to ascertain seroconversion rates in vaccinated populations. The aim of the present study was to standardize the DBS sampling technique for enhanced recovery of critical analytes and to produce quantitative results to compare to data from enzyme-linked immunosorbent assays (ELISA) or hemagglutination assay (HAI)s. This study focused on the feasibility of using DBS in assessing IgG detection using ELISA technology of individuals who were previously vaccinated against measles, varicella, hepatitis A, tetanus and the detection of immune response by microneutralization assay of individuals vaccinated against influenza.

## Materials and methods

### Study population

The study protocol was approved by the Walter Reed National Military Medical Center (WRNMMC) Institutional Review Board in compliance with all applicable federal regulations governing the protection of human subjects. Adults between the ages of 18 and 49 years old were recruited from the Walter Reed National Military Medical Center, a tertiary care medical facility in Maryland. Interested subjects were requested to have previously completed three of the vaccine series being studied and/or natural exposure to the pathogens, and the series/vaccine must have been received at least two weeks prior to enrollment. Individuals were required to be able to provide written informed consent and be beneficiaries of the military medical system. Immunocompromising conditions (such as HIV, cancer, diabetes), or the use of immunomodulatory medications or having undergone treatments likely to affect T or B cell populations within sixty days of enrollment represented exclusion criteria. Pregnant women or women who thought they may be pregnant were also excluded. Documentation of vaccinations for measles, varicella, hepatitis A and tetanus was obtained through the Military Health System’s electronic medical record system. All enrolled subjects had previously received vaccines against all five pathogens being studied. Subjects demonstrated a wide and variable range of time intervals from time of vaccination across the vaccines of interest. The subjects were all recruited to participate on a volunteer basis and were recruited between February 2018 and June 2018 and were compensated for their participation. Study flyers were distributed to various clinics in the hospital (displayed in the clinic lobbies) and on public bulletin boards, as permitted by the facility for recruitment. Interested subjects contacted the Clinical Trial Assistant or the Principal Investigator if interested in being screened. Because this cohort of subjects primarily consisted of active duty military members, veterans or military family members, and vaccinations are highly accessible to this population, the cohort was highly immunized. This research cohort can be considered to be representative of a similarly vaccinated adult population. Detailed demographic information for enrolled subjects is included in the Results and Discussion section. Written informed consent was obtained from all participants and the study was approved by the WRNMMC Institutional Review Board.

### Blood specimen preparation

Paired serum and whole-blood samples were collected from the subjects. The whole-blood samples were collected by venipuncture into heparinized Vacutainer tubes. Sera were collected from the clotted blood samples, aliquoted, and stored at −20°C until tested in the laboratory. For dried blood spot (DBS), whole blood samples were collected by finger prick onto Whatman903 protein saver cards (GE Healthcare Europe, Sigma-Aldrich, catalog # WHA10531018). The cards were allowed to dry at room temperature for two to four hours and stored at -70°C in gas-impermeable zipper bags containing one to two desiccant sachets until assayed.

### Elution and quantitation of IgG from DBS

Three 6-mm discs were punched from each DBS card into a 24 well plate containing 750 μl of ELISA assay sample dilution buffer. The plate was incubated on a shaker at room temperature for 30 minutes and then at 4°C overnight and then placed on an orbital shaker at room temperature again for 30 minutes just prior to the ELISA assay evaluation. The paired sera samples and DBS eluates were tested for IgG antibody levels using the following commercial ELISA assay kits; 1) Anti-measles Antibody (Genway Biotech, catalog # GWB-984A72), 2) Anti-tetanus Ab (Genway Biotech, catalog # GWB-FCBEAB), 3) Anti-Hepatitis A Ab (Creative Diagnostics, catalog # DEIA007) and 4) Anti-Varicella Ab (Genway Biotech, catalog # GWB-BQK23C). ELISA assays were performed according to the manufacturers’ instructions in all four assays without modification, the endpoint analysis parameter was absorbance at 450 nm. Serum and DBS samples were diluted 1:100 for tetanus and measles, for HAV sera was tested undiluted and DBS at 1:21 while VZV was tested at 1:50 dilution. The ELISA kit assay sample buffer was used as the diluent and eluate dilutions were approximated to match sera dilutions.

### Influenza hemagglutination inhibition assay

Serum and DBS samples were screened for anti-HA influenza antibody titers against the influenza strains A/Michigan/45/2015 (International Reagent Resource, catalog # FR-1428), A/Hong Kong/4801/2014 (International Reagent Resource, FR-1453), A/Singapore/INFIMH-16-009/2016 (International Reagent Resource, catalog # FR-1590), A/Singapore/GP1908/205(IVR1980A) and A/Singapore/INFIMH-16-0019/2016 (VR186) (Obtained directly from the CDC, Atlanta Ga., no catalog # is available), B/Phuket/3073/2013 (International Reagent Resource, catalog# FR-1365), and B/Brisbane/60/2008 (International Reagent Resource, catalog# FR-177) by HAI assay, established by Centers for Disease Control and Prevention [17]. The most commonly used HAI assay is based on the ability of the anti-influenza antibodies to bind to the influenza virus and hence prevent the attachment of influenza virus to the red blood cells and hence inhibiting hemagglutination. Receptor destroying enzymes (RDE) are added to serum samples and incubated at 37°C for 18–20 hrs. Two-fold serial dilutions of the RDE-treated serum samples were prepared in 96-well plates and known titers of the test virus was added to each well and incubated at room temperature for 30 minutes. After the 30 minutes incubation, standardized turkey RBC’s (Lampire Biological Laboratories Inc., catalog # 7209403) were added to all wells and incubated for another 30 minutes. After 30 minutes, the plates were observed for agglutination of RBCs. HAI titer is expressed as the reciprocal of the highest dilution of serum that completely inhibited hemagglutination [17]. It is recognized that HAI assay can lack reproducibility and consistency due to difficult to standardize reagents such as RBCs and a number of recent influenza strains, as a result of certain specific mutations, have been shown to have reduced ability for hemagglutination. Therefore, an alternative, more sensitive MN assay was performed, which utilizes the ability of virus-specific antibodies to neutralize the influenza virus and directly prevent the infection of cells in culture.

### Microneutralization assay

The Influenza microneutralization (MN) assay is based on the protocol established by World Health Organization [18]. Sera samples were tested against Influenza strains A/Michigan/45/2015 and A/Hong Kong/4801/214 for the presence of antibodies against hemagglutinin protein. Briefly, two-fold dilutions of serum samples were prepared, each dilution mixed with known amount of the test virus (typically 100 x TCID_50_) and incubated at 37°C for 1 hr. The diluted sera and virus mixture were then used to infect MDCK cells (Sigma-Aldrich, catalog # 05071502) in 96 well plates and the plates are incubated at 37°C for 18–20 hours. After incubation, the virus infected cells are detected by ELISA (for the presence influenza nucleoprotein). Based on the ELISA values of cell controls and virus controls, 50% reduction in infections for the samples are determined and the results are expressed as the reciprocal of the dilution resulting in 50% reduction in infection.

### Stability study

**Fig 1:**
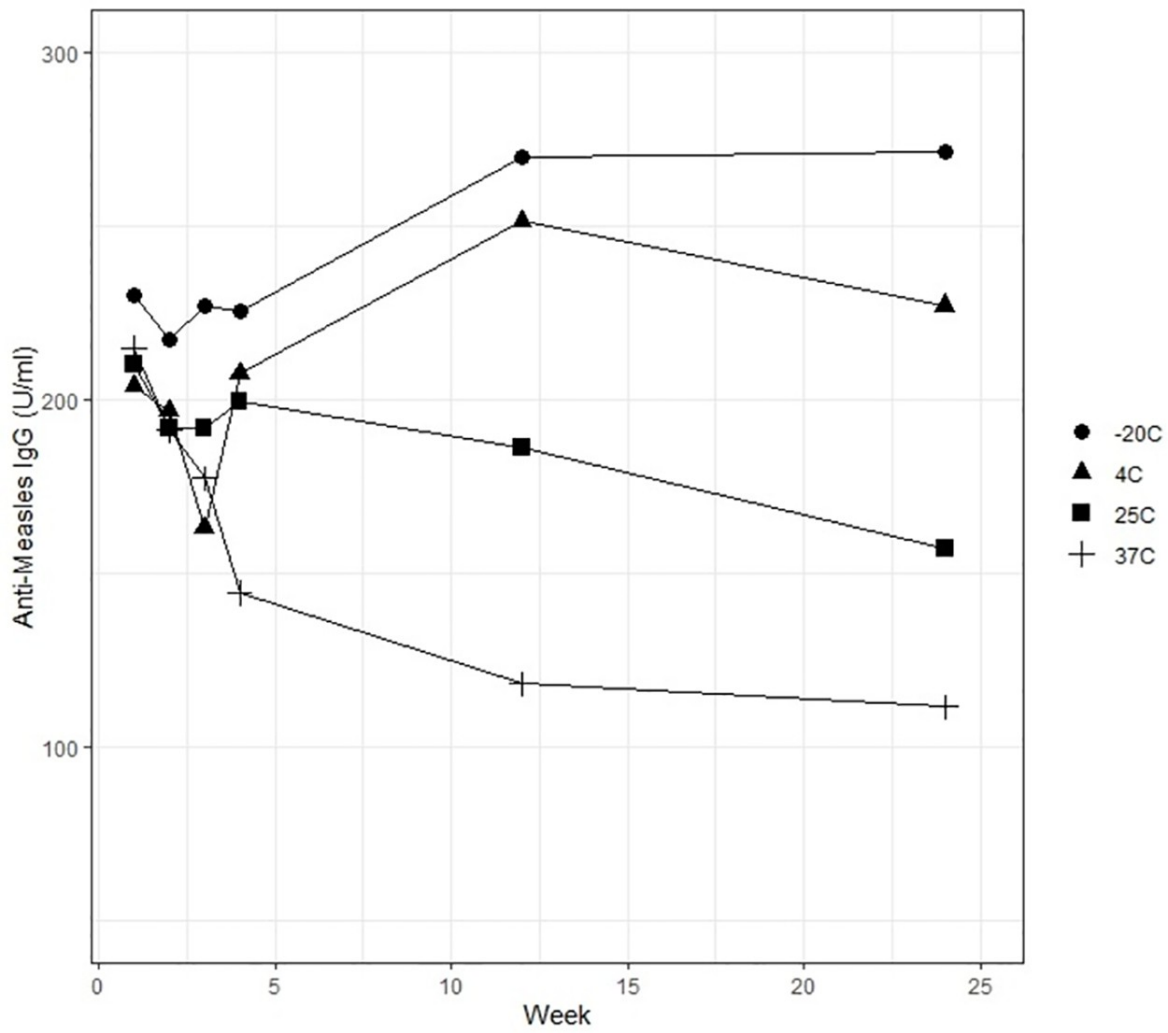
Stability of the blood samples on DBS cards stored under various temperatures for 25 weeks as measured by the titers of anti-measles antibody (IgG) at various time and temperature points.

Measles immune blood stored on DBS cards under varying temperatures was used as a model to assess the stability of IgG. Serum and red blood cell specimens were de-identified, commercially purchased specimens. Equal volumes of measles immune serum (Golden West Biologicals Inc., catalog # D4220) and washed human erythrocytes “O” type (golden West Biologicals Inc., catalog # SD1040.01) were mixed together as the blood spot specimen preparation. Non-immune serum served as the negative control. Fifty microliters of the simulated immune blood was pipetted onto the Whatman 903 protein saver cards. The cards were allowed to dry at room temperature for 2 to 4 hours and stored as mentioned above. For the stability study, duplicate cards were stored at -20°C, 4°C, 25°C and 37°C with desiccant sachets. Eluates from the cards were tested at 1, 2, 3, 4, 12, and 24 weeks using ELISA assays. Mean IgG concentration (U/ml) of the duplicate measures for each temperature and time point is reported in Fig 1.

### Statistical analysis

The detection of antibodies directed against measles, varicella, tetanus and Hepatitis A in serum was used as the gold standard for assessment of sensitivity, specificity, positive predictive values (PPV) and negative predictive values (NPV) for the DBS assays. Bland-Altman plots were used to evaluate for variation in the ratio versus the mean of serum optical density (OD) and DBS OD.

**Fig 2:**
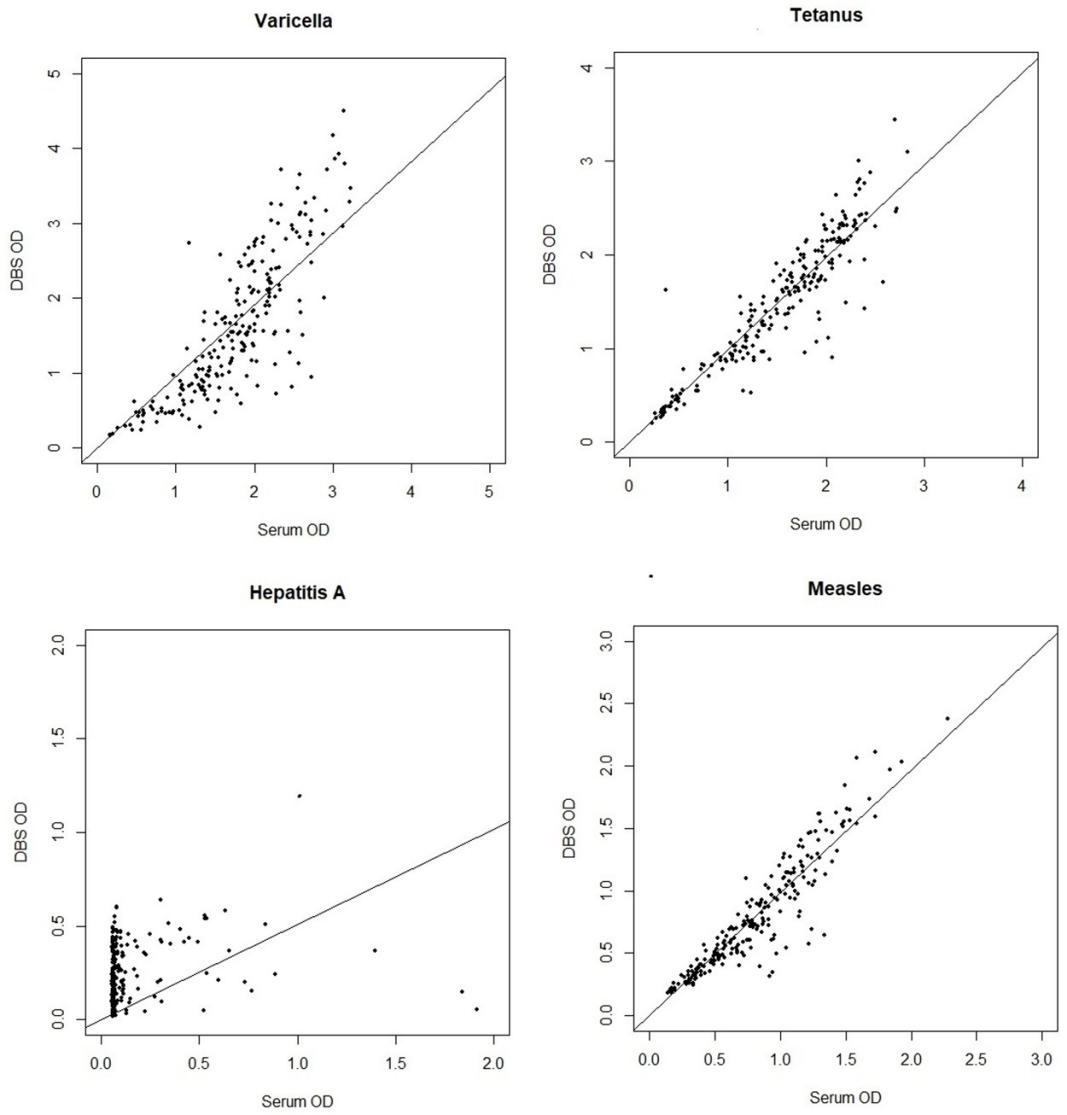
Correlation of IgG antibody levels detected by DBS sampling to IgG antibody levels detected by serum sampling for measles, hepatitis A, tetanus and varicella zoster.

Because DBS OD was proportionally lower than serum OD across the range of concentrations, adjustment factors were calculated to relate DBS to serum values using the regression-based approach described by Riddell et al. [19]. Adjustment factors were 1.36 for measles, 1.28 for tetanus and 1.90 for varicella. An adjustment factor could not be calculated for Hepatitis A because of the nonlinear relationship of serum and DBS OD values for this analyte only. Cutoff values for positivity were defined by the assay protocol and applied to serum OD and adjusted (for measles, tetanus, and varicella) or unadjusted (Hepatitis A) DBS OD. Kappa values and correlation coefficients were calculated to describe concordance between results obtained for DBS and corresponding serum samples. R-squared values were estimated using linear regression models of DBS OD on serum OD with the intercept constrained to the origin (Fig 2). Separate linear regression models were used to evaluate the association of time from the relevant vaccination to sample collection (years) with the serum OD values and with the ratio of DBS to serum OD. Statistical analysis was performed using R studio [20].

## Results and discussion

Two hundred and twenty adult subjects were enrolled in the study. The median age was 26 years with a range of 18–48 years; Fifty seven percent were male (n = 126). Among the participants, white non-Hispanics were the most highly represented (50.4%), followed by black non-Hispanics (24%), Asians (10.4% and white Hispanics (10%). The median (range) time from vaccination to sample collection was 3 years (0–34) and was similar for each vaccine.

Paired sera and DBS eluates from the 220 subjects were tested for anti-measles, anti-tetanus, anti-varicella and anti- Hepatitis A antibodies using commercial ELISA assay kits, and the OD values for these ELISA assays are shown in S1 Fig. DBS results were compared to the corresponding serum results, using the serum as the gold standard. Bland Altman plots showed no substantial variation in the ratio versus mean OD across the range of mean OD values. Table 1 summarizes DBS assay parameters and agreement with serum OD, for adjusted (measles, tetanus and varicella) and unadjusted (Hepatitis A) DBS OD.

**Table 1:** Statistical comparison between serum and DBS samples.

Pathogen	Sensitivity (95% CI)	Specificity (95% CI)	Positive Predictive Value (95% CI)	Negative Predictive Value (95% CI)	Correlation Coefficient Value	Kappa Value (95% CI)
**Measles**	0.92 (0.86, 0.95)	0.96 (0.87, 1.00)	0.99 (0.95, 1.00)	0.78 (0.66, 0.87)	r = 0.93	0.81 (0.68, 0.94)
**Tetanus**	1.00 (0.99, 1.00)	--	1.00 (0.98, 1.00)	--	r = 0.91	--
**Varicella**	0.94 (0.90, 0.97)	1.00 (0.54, 1.00)	1.00 (0.98, 1.00)	0.33 (0.13, 0.59)	r = 0.82	0.48 (0.37, 0.59)
**Hepatitis A**	0.31 (0.25, 0.38)	0.75 (0.19, 0.99)	0.99 (0.92, 1.00)	0.02 (0.00, 0.06)	r = 0.14	0.00 (-0.02, 0.03)

Sensitivities for measles, tetanus, and varicella ranged from 92–100% and specificities for measles and varicella ranged 96–100%. Specificity and NPV were not calculated for tetanus because all DBS and serum values were above the cut-off recommended by the assay protocol. For hepatitis A, sensitivity was 31% and specificity 75%. In unadjusted linear regression models, greater time from vaccination (years) was associated with lower tetanus serum OD (Beta (SE) = -0.03 (0.01); p = 0.04). Time from vaccination was not associated with OD values for other analytes or with the ratio of DBS to serum OD for any analyte. The lack of significant variation from time of vaccination lends further clinical applicability and relevance, extending the utility of the DBS method.

Testing of the paired serum and DBS eluate samples were tested for anti-influenza antibody titers which were achieved from testing the serum samples however the DBS eluates showed no response by both HAI and MN assays. This suggests that the protocol used in this study for antibodies elution from the DBS is not well-suited for the detection of anti-influenza antibodies.

The DBS sample stability study was conducted in order to observe any changes in ELISA absorbance values under different time and temperature conditions. The data indicated that DBS cards containing measles antibodies were stable for four weeks when stored at room temperature and for six months at 4°C. This finding is consistent with the study by Condorelli et al., [21] who showed that DBS cards can be stored at room temperature for 15 days and at 4°C for several months without processing, which is advantageous in areas with limited cold chain capacity and laboratory facilities. However, the samples stored at 37°C showed a substantial decline after week 4, and the 25°C samples demonstrated a modest decline after week 12. Samples stored at -20°C and 4°C showed stability through 6 months, consistent with previous reports that DBS samples may be best stored at temperatures lower than room temperature for some analytes [22].

As depicted in Table 1 above and displayed in Fig 2, there is strong correlation and concordance between DBS sampling and serum testing for measles, varicella and tetanus. This strong correlation between these two analytical methods may be at least partly explained by the strong immunogenicity of the live-virus vaccines, measles and varicella, and the more robust, T-cell dependent humoral response generated by tetanus vaccination. Given this correlation, future study should be directed to more specifically compare DBS results to established vaccine correlates of protection. DBS OD was highly correlated with serum OD for antibodies to measles (r = 0.93), varicella (r = 0.82) and tetanus (r = 0.91).

These findings are concordant with other studies demonstrating that DBS are feasible alternatives to venous blood as samples in investigations for the detection of anti-measles antibodies [19, 23, 24].

Data from this study support the feasibility of using DBS for the detection of anti-tetanus antibodies. The results of the current study demonstrated that the correlation coefficient between the paired serum and DBS eluates relative to the detection of anti-tetanus antibody was high (r = 0.91). This finding is in accord with the study of Nikoletti et.al. [25], which showed that there was no significant difference between DBS eluates and sera samples with respect to the detection of anti-tetanus antibodies.

In the current study, the correlation coefficient between the sera and DBS samples with respect to the detection of antibodies directed against varicella zoster was high (r = 0.82). The results of this study are in agreement with the work of Higgins et al. [26]. Using a chemiluminescent multiplexed immunoassay with reference serum samples, these investigators showed that the sera and DBS samples from vaccinees displayed equivalent ability to detection of anti-varicella antibodies. This finding suggests that DBS may be employed in the detection of the latter.

In the present study, the ELISA data showed that the correlation coefficient between paired serum and DBS eluates regarding the detection of anti-Hepatitis A antibody was very low (r = 0.14) and the sensitivity was 31%. This finding is at variance with the work of Melgaco et al. [27] who showed in a sero-epidemiological study, a strong correlation between paired plasma and DBS samples for the detection of anti-Hepatitis A antibodies reporting the sensitivity and specificity as 100%. Moreover, Gil et al., [28], in a serosurvey of hepatitis A immunity, demonstrated that the DBS eluates had sensitivity and specificity of 91 and 99%, respectively. The discrepancy between the former and the latter two studies may be ascribed to the ELISA assay methodology and the antibody response in research subject samples. Although we observed good correlation between DBS and sera samples for antibodies against tetanus and varicella zoster, we did not detect the presence of any anti-influenza antibody in the DBS eluates. It is possible that the protocol used for eluting antibodies from DBS is not suitable for anti-influenza antibodies. Similarly, an issue with antibody extraction method may also explain the low correlation observed in this study for the anti-Hepatitis A antibodies. This warrants further studies to evaluate and optimize the protocol used for eluting antibodies from DBS.

## Conclusions

Utilization of DBS sampling assays were sensitive and very specific in detecting antibodies against measles, and varicella and highly correlated with serum for these analytes. The testing from DBS eluates of subjects previously immunized against tetanus also demonstrated a high sensitivity. Furthermore, DBS cards displayed long-term stability for some antibodies under refrigeration and stored at -20°C. This is encouraging, especially given the marked variability of subjects’ time from vaccination to testing and these data further strengthen the utility of the DBS testing approach in the assessment of vaccination exposure to different pathogens.

As has been previously noted in the literature, DBS offers a means of readily obtaining samples that can withstand even significant variables with regard to environmental, storage, and shipping conditions. Mitigation strategies to optimize sample collection and transport conditions of DBS cards and better control temperature, humidity and environmental impacts include the use of coolers and desiccants, and bagging the cards. This characteristic of the sample collection method makes it particularly attractive for remote or austere environments where medical infrastructure or other such limitations require a resilient testing platform. Similarly, the high-throughput offered by DBS creates a scalability that has the potential to flexibly serve disparate populations such as deploying service-members and in the context of a public health need for mass testing. The strong correlation of DBS and serum results for measles, tetanus, and varicella point to the need for continued and larger studies to further explore this medium in assessing antibody titers in response to specific vaccinations. Therefore, it is important to determine supplemental methods for sample collection assessment and monitoring of seroconversion.

This research further contributes to the search for cost-effective, convenient, reproducible and sensitive assessment methods in this field. This established method of using DBS to assess the immunogenicity against the vaccines may lead to a follow up study to determine the requirement for booster doses in individuals traveling to these infectious endemic regions as well as applicability of use in other pathogens.

## Supporting information

S1 Fig Quantitation of optical density (OD) for measles, hepatitis A, tetanus and varicella zoster as measured using commercially available ELISA kits on DBS and corresponding serum samples. (TIF)

S1 Data (XLSX)
