# Supplementary figures and images for: The use of dried blood spot cards to assess serologic responses of individuals vaccinated against measles, hepatitis A, tetanus, influenza and varicella zoster
Source: PLoS One. 2022 Mar 24;17(3):e0265813. doi: 10.1371/journal.pone.0265813 (PMC8947131; doi:10.1371/journal.pone.0265813)

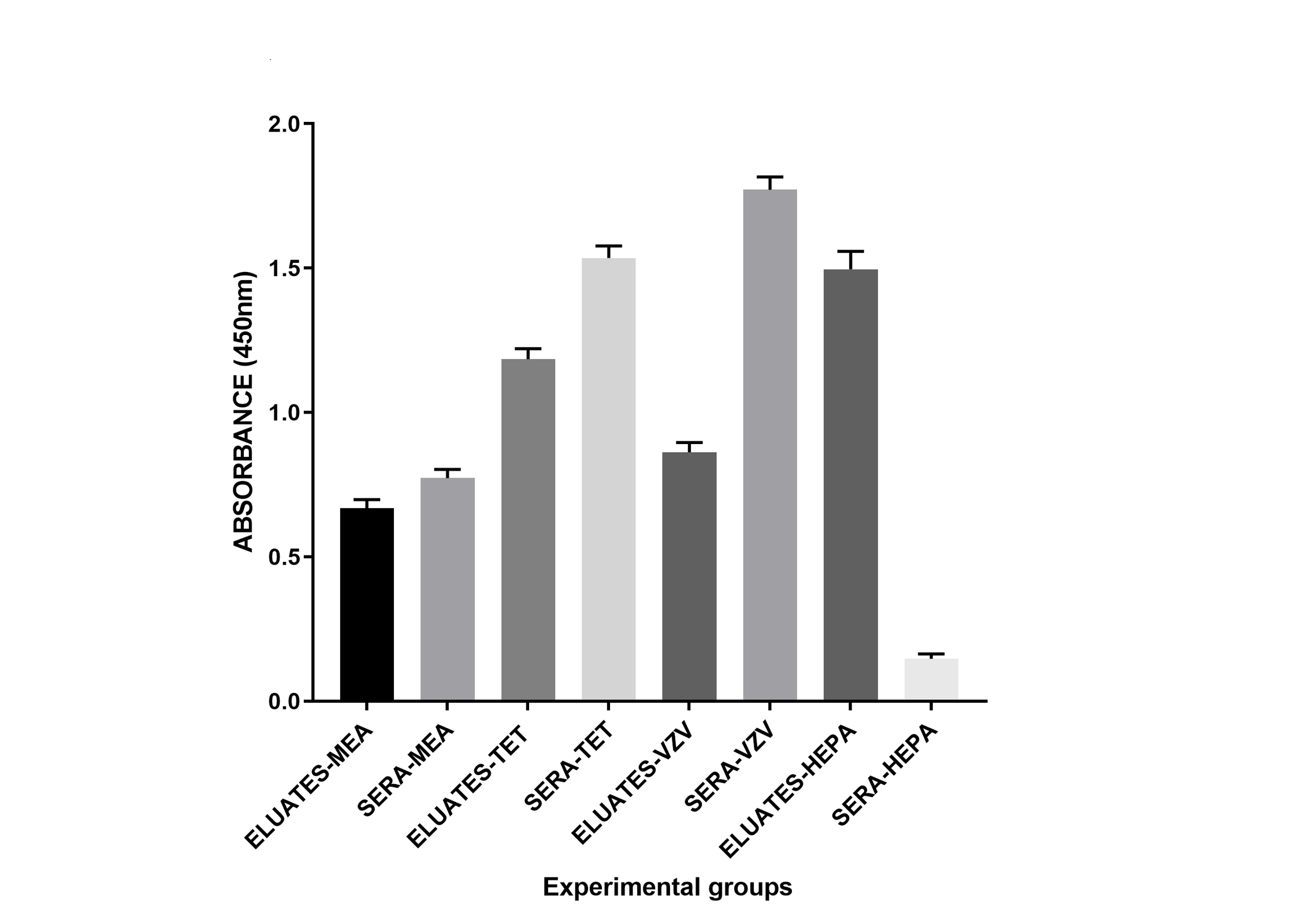

Supplement: S1 Fig — (TIF) [file pone.0265813.s001.tif]
